# Nutritional Intake and Timing of Marathon Runners: Influence of Athlete’s Characteristics and Fueling Practices on Finishing Time

**DOI:** 10.1186/s40798-024-00801-w

**Published:** 2025-03-16

**Authors:** Rubén Jiménez-Alfageme, Florencia Pino Garrone, Nidia Rodriguez-Sanchez, David Romero-García, Isabel Sospedra, Daniel Giménez-Monzó, César Iván Ayala-Guzmán, José Miguel Martínez-Sanz

**Affiliations:** 1 Physiotherapy Department, Faculty of Health Sciences, University of Vitoria-Gasteiz EUNEIZ, 01013 Vitoria-Gasteiz, Spain; 2 Genetic Center Jorge Dotto, Jorge Dotto, Uruguay; 3 Physiology, Exercise and Nutrition Research Group, University of Stirling, Stirling, Scotland, UK; 4 Faculty of Health Sciences, University of Alicante, 03690 Alicante, Spain; 5 Nursing Department, Faculty of Health Sciences, University of Alicante, 03690 Alicante, Spain; 6 Department of Community Nursing, Preventive Medicine and Public Health and History of Science Health, University of Alicante, 03690 Alicante, Spain; 7 Health Department, Iberoamerican University, Mexico City, 01219 Mexico

**Keywords:** Sport nutrition, Fluid intake, Competition, Carbohydrates, Endurance athlete

## Abstract

**Background:**

Endurance athletes’ competitions have increased over the decades and marathon races are becoming increasingly popular. Proper nutrition is critical for optimal performance and long-term health in marathon athletes. This study aimed to investigate runners’ nutritional intake, especially fluids, food, and supplements, competing in the Seville Marathon. A descriptive and cross-sectional study was carried out to obtain information on the consumption of liquids, food, and supplements. A total of 160 runners (aged 42.2 ± 7.3 years) who were primarily men (87.5%) who participated in the 2022 Seville marathon took part in the study.

**Results:**

There was no significant difference (*p* > 0.050) between marathon finish time (from 2 h 12 min to 5 h) or sports experience and fluid, carbohydrates (CHO), sodium, and caffeine intake pre- and post-competition. However, according to the results obtained, the athletes who met CHO intake recommendations during the competition (60–90 g/h) were more likely to finish the marathon in less than 180 min (*p* = 0.035).

**Conclusions:**

The intake of CHO (35 ± 17 g/h), sodium (192 ± 150 mg/h) and caffeine (57 ± 49 mg/h) was low compared to the current recommendations during the competition. The intake of fluids (466 ± 279 mL/h) was at the lower limit of recommendations. Most athletes did not receive nutritional counselling by a sport dietitian, which may explain why athletes failed to meet specific nutrient recommendations. Future investigations with a larger sample size are warranted to assess the relationship between dietary intake and finish time.

## Key Points

- It was observed that athletes who met the recommendations for CHO intake during the competition were more likely to finish the marathon in less than 180 min (p = 0.035).
- Post-competition intake reached the recommended value (0.8–1 g/kg/h) for CHO intake with a value of 0.8 ± 0.5 g/kg but did not meet the minimum recommended (0.3 g/kg/h) for protein with a total of 0.1 ± 0.2 g/kg.
- Fluid intake (466 ± 279 mL/h) was slightly higher compared to other sports such as triathlon (422 ± 176 mL/h) and trail running (447 ± 232 mL/h), but sodium intake (192 ± 150 mg/h) was significantly lower (270 ± 188 mg/h for triathletes and 289 ± 166 mg/h for trail runners).
- The use of the Nutritional Intake Questionnaire for Endurance Competitions (NICEQ) allowed estimating, describing and comparing the type of liquid, food or supplement consumed; the nutritional intake of kcal, macronutrients, sodium and caffeine; incidence and causes of gastrointestinal complaints and their relationship with food intake; and compliance with the dietary-nutritional recommendations for the hour before, during and the hour after the competition.

## Background

Endurance sports, such as cycling, running, triathlon, mountain biking, and cross-country skiing [1], have experienced significant growth among amateur and professional athletes over the decades [2]. According to the International Association of the Athletics Federations (IAAF), long-distance events comprise races on foot between 5000 m and 42.195 km in the marathon [3].

When it comes to marathon runners (MR), carbohydrates (CHO) are the most critical energy substrate for training and competition [4]. Although energy for adenosine triphosphate (ATP) production can be derived from CHO and fats at the same time, as exercise intensity increases (e.g.,65–80% V.O_2_max), MR rely largely upon CHO as the main fuel source [5, 6]. Glycogen and blood glucose are an essential fuel for energy production in the contracting skeletal muscles [7]. However, endogenous glycogen stores are limited (liver: ~ 80–100 g and skeletal muscle: 300–400 g) and its depletion is associated to fatigue during exercise [8, 9].

There are well-established nutritional guidelines for endurance athletes like MR, with CHO being the most closely studied [2, 5]. Both recreational and competitive endurance runners are recommended to consume an adequate CHO, protein, liquids, and sodium intake before, during, and after high-intensity training sessions or competition [9].

Given that starting exercise with low glycogen stores may influence work capacity [4], consuming CHO before exercise is beneficial to maximise glycogen stores and, consequently, enhance endurance performance [10, 11]. It has been demonstrated that 1 h of high-intensity exercise can deplete up to 70% of muscle glycogen stores [12]. Therefore, guidelines recommend that athletes consume 1–4 g of CHO per kg body mass 1–4 h before exercise to increase muscle glycogen levels [13].

According to pre-exercise protein intake recommendations, MR are advised to consume 0.3 g/kg depending on gastrointestinal (GI) tolerance due to the significant muscle catabolism that may occur during the race [2]. Regarding pre-race fat intake, athletes should limit high-fat foods to avoid GI discomfort [2]. To improve endurance performance a common strategy is the use of a caffeine dose of 3–6 mg/kg in the 30–90 min prior to exercise to maximise effects [14]. Indeed, there is new evidence that confirms the benefits of caffeine as an ergogenic aid for endurance events [15]. Previous studies in endurance sports that have evaluated the caffeine intake generally reflect that athletes do not ingest adequate amounts, especially in runners [16–19].

CHO consumption during exercise has an ergogenic effect on endurance performance and also a positive influence on immune function [20]. The American College of Sports Medicine (ACSM) guidelines for CHO consumption are per hour of exercise and recommends consuming 60 g CHO/h for the first 2.5 h of exercise and to consume up to 90 g CHO/h when exercise lasts more than 2.5 h [13]. Indeed, recent studies demonstrated that consuming more than 120 g/h could be beneficial for neuromuscular fatigue and recovery, but achieving this intake requires gut adaptation [9, 21]. Despite the well-documented benefits of CHO consumption, endurance athletes do not tend to meet the recommendations [18, 22, 23]. As far as sodium intake is concerned, the ACSM recommendation is 300–600 mg/h during prolonged exercise > 2 h to reduce the risk of dehydration and prevent hyponatremia [24]. As to caffeine consumption, small doses every 1–2 h may provide benefits for performance [2]. Additionally, the ACSM recommends drinking 400–800 mL/h of fluid depending on factors such as sweat rate and environmental conditions [25].

Post-exercise CHO consumption is one of the most important nutritional strategies to speed recovery. It has been shown that consuming CHO (0.8–1 g/kg/h) immediately after exercise resulted in higher replenishment of glycogen stores [26]. Indeed, when CHO cannot be consumed in sufficient amounts, adding ≥ 0.3 g/kg/h of protein could be beneficial to enhance glycogen resynthesis and also promote skeletal muscle synthesis, as well as adding small amounts of caffeine [2, 14]. To replace fluid, typical guidelines recommend consuming 150% of fluid lost based on body weight together with sodium content (> 60 mmol/L) to retain more fluid [2].

Despite the large number of MR around the world and the importance of nutrition in race success, little is known regarding athletes intake practices and most studies have investigated the nutritional intake during the competition mainly CHO [18]. Despite CHO intake being positively associated with faster running times [19, 23], consuming the adequate amount during the race could be a challenge for endurance athletes since they commonly tend to suffer from GI symptoms [27].

Considering the high nutritional demands associated with marathon races, athletes should consume the recommended amount of macronutrients and fluids pre-, during and post-competition to support health, performance and recovery. The few studies that investigated dietary intakes in endurance athletes, primarily focused on the intake during the event [16, 18, 19]. To address gaps in the previous literature, the present study aimed to examine the dietary intake of MR before, during and after the race, and to determine if descriptive and training characteristics and pre-competition or during competition intake predict marathons’ finishing time independently of the age and sex of participants.

## Methods

### Design

A descriptive and cross-sectional study to investigate the liquids, food, and supplements consumption in endurance runners participating in the Seville marathon was carried out. The sample size calculation was performed with Rstudio software (version 3.15.0, Rstudio Inc., Boston, MA, USA). The significance level was set a priori at p = 0.05. The standard deviation (SD) was set according to the carbohydrate intake (g/h) data from previous studies on ultra marathon runners (SD = 15.2) [18]. With an estimated error (d) of 2.35, the sample size needed was 160 subjects. The study population was selected by non-probabilistic, non-injury, convenience sampling among organizing institution of the Seville Marathon 2022. This study was approved by the ethics committee of the University of Alicante with the file number UA-2022-02-01 and followed the World Medical Association codes and Declaration of Helsinki for research in humans. In addition, the study design as well as the development of the manuscript followed the STROBE statement [28].

### Competition, Participants and Sample Size

The competition was a marathon consistent in a 42.195 km race in a semi-self-sufficient manner where 15 aid stations located approximately every 3 km, offering the usual food and beverages typically provided in this type of race to meet the needs of all runners. Participants could choose between liquids, solids, and gels (https://www.zurichmaratonsevilla.es/zms-avituallamientos-wcs-y-asistencia-medica). Additionally, each athlete could bring their own food and drinks.

The sample consisted of 160 marathon runners who were aged 42.2 ± 7.3 years old. Among them, 87.5% were men and 12.5% were women, and all were adults (≥ 18y). The participants had competed in regional, national, and international competitions for at least two years and had not suffered from any injuries or illnesses in the six months prior to the survey. The participants’ competitive level varied from regional competitions (held at the provincial and regional level) to national competitions (held throughout Spain) and international competitions (held worldwide). Table 1 provides information on the age, basic anthropometric characteristics, years of sports experience and finishing time of marathon of the study participants.

**Table 1:** Descriptive characteristics of a sample of marathon runners

	Total		Marathon finishing time		
	(n = 160)		121–180 min (n = 37)	181–240 min (n = 99)	> 241 min (n = 24)
	n	%	%	%	%
*Sex*					
Female	20	12.5	0.0	13.1	29.2
Male	140	87.5	100.0	86.9	70.8
*Federated athlete*					
No	116	72.5	56.7	71.7	100.0
Yes	44	27.5	43.2	28.3	0.0
*Sports experience*					
PARL	99	63.4	61.1	61.2	77.3
NINL	57	36.6	38.9	38.8	22.7
*Sports modality*					
Triathlon	13	8.3	16.2	5.1	8.7
MRRC	19	12.1	10.8	11.3	17.3
Marathon	125	79.6	73.0	83.5	73.9
*Exercise frequency (s/w)*					
1–3	30	18.7	5.4	16.2	50.0
4–6	108	67.5	64.9	74.7	41.7
≥ 7	22	13.8	29.7	9.1	8.3
*Exercise time (h/w)*					
1–5	54	33.7	10.8	40.4	41.7
6–7	46	28.7	21.6	28.3	41.7
≥ 8	60	37.6	67.6	31.3	16.6
*DTS*					
No	95	60.1	54.1	60.8	66.7
Yes	63	39.9	45.9	39.2	33.3
		Mean ± SD	Mean ± SD	Mean ± SD	Mean ± SD
Age (y)		42.2 ± 7.3	39.4 ± 5.2	42.4 ± 7.4	45.4 ± 8.0
Height (m)		1.7 ± 0.1	1.8 ± 0.1	1.7 ± 0.1	1.7 ± 0.1
Weight (kg)		70.1 ± 9.7	69.8 ± 8.5	69.3 ± 9.1	74.3 ± 13.1
BMI (kg/m^2^)		22.9 ± 2.1	22.3 ± 1.8	22.7 ± 1.9	24.5 ± 2.5
TSM (y)		9.2 ± 6.2	8.6 ± 4.8	9.2 ± 6.4	9.8 ± 7.5
Exercise frequency		4.9 ± 1.5	5.9 ± 1.6	4.7 ± 1.3	3.8 ± 1.4
Exercise time (h/w)		7.2 ± 3.6	8.5 ± 2.7	6.9 ± 3.9	6.2 ± 2.9

*BMI* body mass index; *LW/NW* low and normal weight; *OW/OB* overweight or obese; *PARL* provincial, autonomous, or regional level; *NINL* national or international level; *MRRC* Mountain’s race and road cycling; *DTS* double training session; *TSM* time in sports modality; *SD* standard deviation; *h/w* hours per week

### Instruments

Fluid, food, and supplement intake, as well as the incidence of gastrointestinal complaints in endurance competitors were collected using a validated online self-administered NIQEC (Nutritional Intake Questionnaire for Endurance Competitions) [29]. It includes 50 questions divided into 5 specific sections: (1) sociodemographic factors; (2) sports data; (3) food, liquid, and supplement intake one hour before, during, and one hour after the race; (4) gastrointestinal symptoms; and (5) dietary–nutritional planning of the test. The coding of the variables and the estimation of energy and macronutrients was carried out by a trained sport dietitian, using the Spanish Database of Food Composition [30] and the data sheets of each sports supplement consumed by the athletes following the guide to perform nutritional estimation based on the NIQEC developed by the authors of the questionnaire [31]. The questionnaire can be consulted in its user manual [32].

### Procedure

Athletes who were invited to participated in the study were sent an electronic informed consent document by the Zurich Marathon of Seville 2022 organisers, which they signed and returned via email. In the consent to participate, the runners were informed about the nature of the study and the possibility of withdrawing from the study at any time via email. Those who agreed to participate received the link to the NIQEC questionnaire with detailed instructions about how to complete it. Participants filled it in voluntarily, telematically, and anonymously.

### Statistical Analysis

The statistical analysis was run with the statistical package STATA 15 (College Station, TX). The mean and standard deviations of the variables were calculated. Normality of the variables was verified by the Kolmogorov–Smirnov test. To analyse differences according to the marathon finishing time, ANOVA or Kruskal Wallis tests were used, depending on the symmetry of the variables. Whereas unpaired T-tests or Mann–Whitney U tests were performed to analyse differences according to sports experience. An alpha level of *p* < 0.05 was established.

The relationship between marathon finishing time with descriptive and training characteristics, and pre-competition and during competition intake were evaluated using Pearson’s correlation coefficients (Table 10). Then, simple linear regression models were estimated in which the dependent variable was marathon finishing time, and the independent variables were those that were related to marathon finishing time (sex, age, body mass index (BMI), exercise frequency and time, and fluids, energy, carbohydrates, and sodium intake during marathon).

Subsequently, only variables that kept significance (*p* ≤ 0.050) in the Pearson’s correlation coefficients and simple linear regression models were retained, and multiple linear regression models were performed using variables that showed correlation and predictive capacity for marathon finishing time (Table 11).

Correlations were interpreted with the rule of thumb [33]. The variance inflation factor (VIF) was calculated to identify multicollinearity in the regression models (VIF > 5), [33]. Akaike’s information criterion (AIC) of each model was computed to contrast models. When the AIC difference was > 10, the model with the lower AIC was considered suboptimal [33].

## Results

Pre competition intake is presented in Tables 2 and 3. According to the most relevant nutrients, athletes consumed 7 ± 4 mL/kg of water, 1 ± 0.5 g/kg of CHO, 4.5 ± 4.5 mg/kg of sodium and 1.5 ± 1 mg/kg of caffeine one hour before the competition. There were no differences of fluids, energy, macronutrient, sodium, and caffeine intake pre-competition according to marathon finishing time or sports experience (Tables 2 and 3). There were no differences between marathon finish time or sports experience and participants who met international recommendations for pre-competition fluid, CHO, and caffeine intake.

**Table 2:** Pre-competition food, fluids, energy, and nutrient intake classified by finishing time

Pre-competition intake	Total		Marathon finishing time			*p*
	(n = 160)		121–180 min (n = 37)	181–240 min (n = 99)	> 241 min (n = 24)	
	n	Mean ± SD	Mean ± SD	Mean ± SD	Mean ± SD	
Fluids (mL/kg)	146.0	7.2 ± 4.1	7.3 ± 4.0	7.0 ± 4.2	5.9 ± 3.6	0.623
Energy (kcal/kg)	125.0	4.6 ± 7.0	3.9 ± 3.9	3.8 ± 3.7	3.7 ± 1.5	0.666
CHO(g/kg)	123.0	0.8 ± 0.6	0.6 ± 0.7	0.6 ± 0.6	0.5 ± 0.3	0.269
Protein (g/kg)	106.0	0.1 ± 0.1	0.1 ± 0.1	1.0 ± 0.1	0.1 ± 0.0	0.613
Fat (g/kg)	100.0	0.1 ± 0.1	0.1 ± 0.1	0.1 ± 0.1	0.1 ± 0.1	0.893
Sodium (mg/kg)	147.0	4.6 ± 4.7	2.4 ± 4.6	3.7 ± 4.9	2.7 ± 4.3	0.336
Caffeine (mg/kg)	73.0	1.3 ± 0.9	1.1 ± 0.4	1.1 ± 1.0	0.9 ± 0.3	0.332

	n	%	%	%	%	*p*
*Fluids*						
< 5 mL/kg	41.0	28.1	20.6	28.9	36.4	0.458
5 – 7 mL/kg	29.0	19.9	17.6	18.9	27.3	
> 7 mL/kg	76.0	52.0	61.8	52.2	36.4	
*Carbohydrates*						
< 1 g/kg	90.0	77.2	65.4	71.8	89.5	0.178
≥ 1 g/kg	33.0	26.8	34.6	28.2	10.5	
*Caffeine*						
< 1 mg/kg	28.0	38.4	28.6	34.8	61.5	0.306
1 – 3 mg/kg	43.0	58.9	71.4	60.9	38.5	
> 3 mg/kg	2.0	2.7	0.0	4.3	0.0	
*Caffeine*						
< 1 mg/kg	28.0	38.4	39.1	30.4	0.713	
1 – 3 mg/kg	43.0	58.9	58.7	65.2		
> 3 mg/kg	2.0	2.7	2.2	4.3

**Table 3:** Pre-competition food, fluids, energy, and nutrient intake classified by sports experience

Pre-competition intake	n	Mean ± SD	Sports experience		*p*
			PARL (n = 99)	NINL (n = 57)	
			Mean ± SD	Mean ± SD	
Fluids (mL/kg)	146.0	7.2 ± 4.1	6.5 ± 3.9	7.3 ± 4.5	0.094
Energy (kcal/kg)	125.0	4.6 ± 3.5	3.8 ± 3.0	4.5 ± 4.2	0.425
CHO (g/kg)	123.0	0.8 ± 0.6	0.5 ± 0.5	0.7 ± 0.7	0.153
Protein (g/kg)	106.0	0.1 ± 0.1	0.1 ± 0.1	0.1 ± 0.1	0.866
Fat (g/kg)	100.0	0.1 ± 0.1	0.1 ± 0.1	0.1 ± 0.1	0.692
Sodium (mg/kg)	147.0	4.6 ± 4.7	2.8 ± 4.6	3.3 ± 5.1	0.641
Caffeine (mg/kg)	73.0	1.3 ± 0.9	1.1 ± 0.8	1.2 ± 1.1	0.449

	n	%	%	%	*p*
*Fluids*					
< 5 mL/kg	41.0	28.1	32.2	21.1	0.312
5 – 7 mL/kg	29.0	19.9	20.0	19.2	
> 7 mL/kg	76.0	52.0	47.8	59.6	
*CHO*					
< 1 g/kg	90.0	77.1	75.3	67.4	0.345
≥ 1 g/kg	33.0	26.8	24.7	32.6	
*Caffein*e					
< 1 mg/kg	28.0	38.4	39.1	30.4	0.713
1 – 3 mg/kg	43.0	58.9	58.7	65.2	
> 3 mg/kg	2.0	2.7	2.2	4.3	

*PARL* provincial, autonomous or regional level; *NINL* national or international level

Regarding the intakes performed during the event, Tables 4 and 5 shows that runners consumed 466 ± 279 mL/h of water, 35 ± 17 g/h of CHO, 192 ± 150 mg/h of sodium and 57 ± 49 mg/h of caffeine. Those who drank less body-weight-relative fluids during competition had more probabilities to finish the marathon in less than 180 min (*p* = 0.011, Table 4). Also, athletes who ate more CHO per hour of exercising had more probabilities to finish marathon race in less than 180 min (*p* = 0.071). There were no differences of total and body-weight-relative energy, protein, fat, sodium, and caffeine intake according to marathon finishing time (*p* > 0.050). There were no differences between marathon finish time and participants who met international recommendations for fluid and caffeine intake during competition (*p* > 0.050), but athletes who met CHO intake (i.e., 30 to 60 g/h) were more likely to finish the marathon in less than 180 min (*p* = 0.035) than the other groups. More experienced athletes consumed slightly more fat than less experienced ones (*p* = 0.045), but there were no differences of fluids, energy, carbohydrates, protein, sodium, or caffeine intake according to sports experience (*p* > 0.050). There were no differences between sports experience and participants who met international recommendations for fluid, CHO, and caffeine intake during competition (*p* > 0.050).

**Table 4:** During competition food, fluids, energy, and nutrient intake classified by finishing time

	Total		Marathon finishing time			*p*
	(n = 160)		121–180 min (n = 37)	181–240 min (n = 99)	> 241 min (n = 24)	
	n	Mean ± SD	Mean ± SD	Mean ± SD	Mean ± SD	
*Competition intake*						
Fluids (mL/h)	154.0	466.1 ± 279.2	499.0 ± 235.4	415.8 ± 303.1	413.3 ± 246.8	0.778
Energy (kcal/h)	156.0	148.2 ± 69.4	159.2 ± 55.6	150.3 ± 74.1	122.7 ± 64.8	0.120
CHO (g/h)	155.0	35.4 ± 16.9	38.9 ± 13.7	35.7 ± 18.0	28.8 ± 15.3	0.071
Protein (g/h)	80.0	0.9 ± 1.3	0.7 ± 1.1	0.3 ± 1.2	0.3 ± 1.7	0.338
Protein (g/kg)	80.0	0.05 ± 0.07	0.03 ± 0.05	0.02 ± 0.07	0.01 ± 1.0	0.732
Fat (g/h)	88.0	1.4 ± 5.1	0.15 ± 1.2	0.08 ± 6.3	0.07 ± 1.3	0.214
Sodium (mg/h)	157.0	192.3 ± 150.3	194.2 ± 139.4	194.2 ± 163.2	181.5 ± 111.1	0.931
Caffeine (mg/h)	69.0	56.6 ± 49.4	43.1 ± 28.4	45.0 ± 52.3	31.4 ± 62.2	0.319

	n	%	%	%	%	*p*
*Fluids*						
< 400 mL/h	71.0	46.1	45.7	45.2	50.0	0.960
400 – 800 mL/h	68.0	44.2	45.7	45.2	37.5	
> 800 mL/h	15.0	9.7	8.6	9.6	12.5	
*CHO*						
< 30 g/h	64.0	41.3	22.2	42.1	66.7	0.035
30 – 60 g/h	76.0	49.0	69.4	47.4	25.0	
60 – 90 g/h	14.0	9.0	8.3	9.5	8.3	
> 90 g/h	1.0	0.7	0.0	1.0	0.0	
*Sodium*						
< 300 mg/h	126.0	80.2	75.0	81.4	83.3	0.647
300 – 600 mg/h	29.0	18.5	25.0	16.5	16.7	
> 600 mg/h	2.0	1.3	0.0	2.1	0.0

**Table 5:** During competition food, fluids, energy, and nutrient intake classified by sport experience

	Total		Sports experience		*p*
	(n = 160)		PARL (n = 99)	NINL (n = 57)	
	n	Mean ± SD	Mean ± SD	Mean ± SD	
*Competition intake*					
Fluids (mL/h)	154.0	466.1 ± 279.2	427.7 ± 249.4	415.8 ± 331.5	0.489
Energy (kcal/h)	156.0	148.2 ± 69.4	141.6 ± 6.2	161.8 ± 11.1	0.088
CHO (g/h)	155.0	35.4 ± 16.9	33.9 ± 1.5	38.6 ± 2.7	0.107
Protein (g/h)	80.0	0.9 ± 1.3	0.3 ± 1.1	0.5 ± 1.4	0.394
Fat (g/h)	88.0	1.4 ± 5.1	0.07 ± 2.1	0.09 ± 8.0	0.045
Sodium (mg/h)	157.0	192.3 ± 150.3	207.6 ± 15.7	170.0 ± 0.19	0.134
Caffeine (mg/h)	69.0	56.6 ± 49.4	34.5 ± 42.0	45.4 ± 61.8	0.169

	n	%	%	%	
*Fluids*					
< 400 mL/h	71.0	46.1	48.4	42.8	0.802
400 – 800 mL/h	68.0	44.2	42.1	46.4	
> 800 mLl/h	15.0	9.7	9.5	10.7	
*CHO*					
< 30 g/h	64.0	41.3	44.3	35.2	0.151
30 – 60 g/h	76.0	49.0	49.5	48.1	
60 – 90 g/h	14.0	9.0	6.2	14.8	
> 90 g/h	1.0	0.7	0.0	1.8	
*Sodium*					
< 300 mg/h	126.0	80.2	76.3	85.7	0.288
300 – 600 mg/h	29.0	18.5	22.7	12.5	
> 600 mg/h	2.0	1.3	1.0	1.8	

*PARL* provincial, autonomous, or regional level; *NINL* national or international level

As for one hour post event intakes, Tables 6 and 7 shows that athlete’s consumption was 15 ± 8 ml/kg of liquids, 1 ± 0.5 g/kg of CHO, 2.5 ± 4 mg/kg of sodium, 0.5 ± 0.2 mg/kg of caffeine. There were no differences between energy, macronutrient, sodium, or caffeine post-competition intake according to marathon finishing time (*p* > 0.050, Table 6). Also, there were no differences between marathon finish time and participants who met international recommendations for post-competition CHO and protein intake (*p* > 0.050). More experienced athletes, compared to less experienced ones, had higher probabilities to eat more energy (*p* = 0.031), protein (*p* = 0.073), fat (*p* = 0.038), and sodium (*p* = 0.005) after the competition, but there were no differences in fluids, CHO, caffeine, or rate of carbohydrates/protein ingestion.after competition according to sports experience (*p* > 0.050,Table 7).

**Table 6:** Post-competition food, fluids, energy, and nutrient intake classified by finishing time

	Total		Marathon finishing time			*p*
	(n = 160)		121–180 min (n = 37)	181–240 min (n = 99)	> 241 min (n = 24)	
	n	Mean ± SD	Mean ± SD	Mean ± SD	Mean ± SD	
*Post-competition intake*						
Fluids (mL/kg)	153.0	14.7 ± 7.9	12.0 ± 6.8	13.6 ± 8.3	14.9 ± 7.5	0.552
Solids (g/kg)	112.0	2.2 ± 1.5	1.9 ± 1.3	1.8 ± 1.6	1.6 ± 1.7	0.523
Energy (kcal/kg)	142.0	4.2 ± 3.2	3.7 ± 2.4	3.7 ± 3.0	3.8 ± 4.7	0.919
CHO (g/kg)	142.0	0.80 ± 0.5	0.65 ± 0.5	0.69 ± 0.5	0.64 ± 0.7	0.912
Protein (g/kg)	119.0	0.12 ± 0.2	0.04 ± 0.1	0.04 ± 0.1	0.03 ± 0.4	0.321
Fat (g/kg)	102.0	0.06 ± 0.1	0.01 ± 0.1	0.01 ± 0.1	0.01 ± 0.1	0.195
Sodium (mg/kg)	153.0	2.4 ± 3.9	1.5 ± 2.2	1.6 ± 2.9	0.7 ± 7.7	0.899
Caffeine (mg/kg)	36.0	0.52 ± 0.2	0.47 ± 0.2	0.46 ± 0.2	0.44 ± 0.1	0.842
Carbohydrates/protein	119.0	20.9 ± 18.8	17.9 ± 17.2	20.9 ± 18.5	25.2 ± 21.9	0.514

	n	%	%	%	%	*p*
*CHO*						
< 0.8 g/kg	80.0	56.3	58.8	56.5	52.2	0.628
0.8 – 1.2 g/kg	36.0	25.3	17.6	25.9	34.8	
> 1.2 g/kg	26.0	18.3	23.5	17.6	13.0	
*Protein*						
< 0.2 g/kg	94.0	79.0	65.5	82.6	85.7	0.117
> 0.2 g/kg	25.0	21.0	34.5	17.4	14.3

**Table 7:** Post-competition food, fluids, energy, and nutrient intake classified by sports experience

Post-competition intake			Sports experience		
			PARL	NINL	*p*
			Mean ± SD	Mean ± SD	
Fluids (mL/kg)	153.0	14.7 ± 7.9	13.0 ± 7.9	13.9 ± 7.9	0.724
Solids (g/kg)	150.0	1.7 ± 1.7	1.7 ± 1.6	1.7 ± 1.5	0.921
Energy (kcal/kg)	151.0	4.0 ± 3.3	3.4 ± 3.1	4.4 ± 3.4	0.031
CHO (g/kg)	151.0	0.7 ± 0.5	0.66 ± 0.5	0.80 ± 0.6	0.244
Protein (g/kg)	150.0	0.09 ± 0.2	0.04 ± 0.2	0.06 ± 0.1	0.073
Fat (g/kg)	150.0	0.03 ± 0.1	0.01 ± 0.1	0.03 ± 0.2	0.038
Sodium (mg/kg)	153.0	2.4 ± 3.9	1.1 ± 4.0	2.0 ± 3.9	0.005
Caffeine (mg/kg)	150.0	0.1 ± 0.2	0.47 ± 0.1	0.46 ± 0.2	0.933
CHO/protein	119.0	20.9 ± 18.8	17.8 ± 16.8	9.7 ± 22.0	0.104

	n	%	%	%	*p*
*CHO*					
< 0.8 g/kg	80.0	56.3	59.5	50.0	0.351
0.8 – 1.2 g/kg	36.0	25.3	25.8	25.0	
> 1.2 g/kg	26.0	18.3	14.6	25.0	
*Protein*					
< 0.2 g/kg	94.0	79.0	81.1	74.5	0.390
> 0.2 g/kg	25.0	21.0	18.8	25.5	

*PARL* provincial, autonomous, or regional level; *NINL* national or international level

As Tables 8 and 9 show, most of the participants followed a special nutrition planning for the event, and most of the athletes (46%) did not receive nutritional advice, but among those who did, most received it from trainers (19%), followed by a sport (19%). Most of the participants ingested some food or drink before, during or after the marathon (91% to 96%). CHO intake prior, during and post competition ranged from 23 to 100 g. From those who ingested CHO one hour prior the sports event, approximately 23 g of CHO were ingested from liquids, 33 g from semi-liquids, and 42 g from solid foods. During competition, athletes ingested 35 g of CHO from liquids, 101 g from semi-liquids, and 25 g from solid foods. After competition, athletes consumed 40 g of CHO from liquids, 14 g from semi-liquids, and 30 g from solid foods.

**Table 8:** Nutrition planning, timing, and counselling of a sample of marathon runners classified by finishing time

	Total		Marathon finishing time			*p*
	(n = 160)		121–180 min (n = 37)	181–240 min (n = 99)	> 241 min (n = 24)	
	n	%	%	%	%	
Special planning	116.0	73.0	86.1	71.7	58.3	0.054
Nutrition planning	130.0	82.3	94.4	78.6	79.2	0.094
*Nutrition counselling*						
Coach	30.0	19.3	13.9	21.9	17.4	0.464
Sport dietitian	29.0	18.7	25.0	19.8	4.3	
Other	25.0	16.1	13.9	15.6	21.7	
None	71.0	45.8	47.2	42.7	56.5	
Pre-competition intake	146.0	91.2	91.9	90.9	91.7	0.981
Competition intake	158.0	98.7	94.6	96.0	100.0	0.538
Post-competition intake	150.0	93.7	91.9	92.9	100.0	0.381

	n	Mean ± SD	Mean ± SD	Mean ± SD	Mean ± SD	*p*
*Pre-competition (1 h, g)*						
CHO from liquids	66.0	23.3 ± 17.9	25.6 ± 20.7	20.5 ± 18.0	15.5 ± 9.3	0.244
CHO from semiliquids	46.0	33.3 ± 28.6	25.4 ± 30.1	19.3 ± 30.8	22.0 ± 11.4	0.384
CHO from solids	87.0	42.0 ± 29.0	37.0 ± 37.4	34.5 ± 28.2	37.9 ± 19.1	0.511
*Competition (g/h)*						
CHO from liquids	84.0	35.0 ± 6.0	21.5 ± 12.9	25.0 ± 34.2	31.5 ± 23.6	0.774
CHO from semiliquids	143.0	100.8 ± 50.0	98.5 ± 41.0	104.6 ± 54.8	89.4 ± 40.8	0.704
CHO from solids	43.0	24.6 ± 20.2	26.8 ± 0.0	20.0 ± 22.9	20.0 ± 14.0	0.685
*Post-competition (1 h, g)*						
CHO from liquids	124.0	40.0 ± 23.8	34.5 ± 30.0	34.0 ± 18.2	39.2 ± 30.8	0.306
CHO from semiliquids	4.0	14.2 ± 5.2				
CHO from solids	98.0	30.2 ± 21.4	20.0 ± 14.9	21.5 ± 24.5	20.0 ± 19.9	0.185

**Table 9:** Nutrition planning, timing, and counselling of a sample of marathon runners classified by sports experience

	Total		Sports experience		
			PARL (n = 99)	NINL (n = 57)	*p*
	n	%	%	%	
Special planning	116.0	73.0	70.7	76.8	0.413
Nutrition planning	130.0	82.3	78.8	87.3	0.191
Nutrition counselling					
Coach	30.0	19.3	18.7	20.0	0.043
Healthcare provider	29.0	18.7	13.5	29.1	
Other	25.0	16.1	19.8	7.3	
None	71.0	45.8	47.9	43.6	
Pre-competition intake	146.0	91.2	90.9	91.2	0.946
Competition intake	158.0	98.7	96.0	98.2	0.435
Post-competition intake	150.0	93.7	96.0	89.5	0.111

	n	Mean ± SD	Mean ± SD	Mean ± SD	*p*
*Pre-competition (1 h, g)*					
CHO from liquids	66.0	23.3 ± 17.9	19.6 ± 16.1	30.0 ± 19.4	0.011
CHO from semiliquids	46.0	33.3 ± 28.6	25.5 ± 27.8	19.4 ± 31.9	0.797
CHO from solids	87.0	42.0 ± 29.0	35.1 ± 24.0	38.5 ± 36.4	0.404
Competition (g/h)					
CHO from liquids	84.0	35.0 ± 6	25.8 ± 25.0	26.6 ± 38.2	0.651
CHO from semiliquids	143.0	100.8 ± 50.0	90.0 ± 44.6	98.0 ± 57.8	0.430
CHO from solids	43.0	24.6 ± 20.2	20.0 ± 18.7	20.0 ± 24.0	0.707
*Post-competition (1 h, g)*					
CHO from liquids	124.0	40.0 ± 23.8	35.0 ± 23.6	34.0 ± 24.6	0.860
CHO from semiliquids	4.0	14.2 ± 5.2			
CHO from solids	98.0	30.2 ± 21.4	31.7 ± 17.5	28.2 ± 27.4	0.070

*PARL* provincial, autonomous, or regional level; *NINL* national or international level; CHO carbohydrates

Participants who had a special nutrition planning for the event had higher probabilities to finishing the race in less than 180 min (*p* = 0.054, Table 8). Moreover, when comparing higher-level athletes competing at the national or international level with those competing only at the local or regional level, it is observed that lower-level athletes receive less nutritional counselling (*p* = 0.043, Table 9). At the same time, more experienced runners ingested more CHO from liquids prior the event (p = 0.011), but less CHO from solids post-competition (*p* = 0.07) (Table 9). Regarding GI complaints, only the 13.42% of the runners reported any discomfort, being the most frequent belching, gas o stomach pain.

Marathon finishing time was negatively correlated with exercise frequency and time (*p* = 0.000) (Table 10), and positively correlated with weight and BMI (*p* = 0.000). Additionally, it was shown that men had shorter finishing times than women.

**Table 10:** Correlation and simple linear regression models in which marathon finishing time is considered the dependent variable, while descriptive and training characteristics, pre-competition, or competition intake are considered independent variables

	Marathon finishing time		Linear regression models			
	r	*P*	α	β	R^2^	SEE
Sex	−0.278	0.000	234.50^*c*^	−28.38^*c*^	7.72	32.65
Age (y)	0.244	0.002	161.60^*c*^	1.14^*b*^	5.96	32.96
Weight (kg)	0.111	0.162				
Height (m)	−0.124	0.117				
BMI (kg/m^2^)	0.287	0.000	104.39^*c*^	4.60^*c*^	8.23	32.56
Time in sports modality (y)	0.027	0.736				
Exercise frequency (s/w)	−0.449	0.000	258.18^*c*^	−9.91^*c*^	20.20	30.36
Exercise time (h/w)	−0.268	0.000	227.77^*c*^	−2.51^*b*^	7.19	32.74
Pre-competition intake						
Fluids (mL/kg)	0.026	0.746				
Energy (kcal/kg)	−0.032	0.688				
CHO (g/kg)	−0.074	0.353				
Protein (g/kg)	−0.029	0.717				
Fat (g/kg)	0.048	0.549				
Sodium (mg/kg)	0.056	0.481				
Caffeine (mg/kg)	−0.016	0.844				
Competition intake						
Fluids (mL/h)	0.002	0.977				
Fluids (mL/kg)	0.241	0.002	195.98^*c*^	0.61^*b*^	5.82	33.29
Energy (kcal/h)	−0.150	0.061	220.82^*c*^	−0.07^*d*^	2.25	33.86
Energy (kcal/kg)	0.126	0.116				
CHO (g/h)	−0.155	0.054	220.95^*c*^	−0.31^*d*^	2.40	33.95
CHO (g/kg)	0.121	0.132				
Protein (g/h)	−0.046	0.686				
Protein (g/kg)	0.038	0.734				
Fat (g/h)	−0.046	0.667				
Fat (g/kg)	−0.020	0.856				
Sodium (mg/h)	−0.019	0.813				
Sodium (mg/kg)	0.144	0.072	203.64^*c*^	0.66^*d*^	2.06	33.79
Caffeine (mg/h)	−0.008	0.946				
Caffeine (mg/kg)	0.134	0.272				

α, intercept; β, regression coefficient; R2, determination coefficient as percentage; *SEE* standard errors of estimate. Coding for sex: 0 = female, 1 = male.

^a^*p* < 0.050, ^b^*p* < 0.010, ^c^*p* < 0.001, ^d^*p* < 0.080

Sex, age, BMI, exercise frequency and time of participants, fluids intake during competition were predictors of marathon’s finishing time (*p* < 0.050). Also, energy, CHO and sodium intake during competition were predictors of marathon’s finishing time (*p* < 0.080). Exercise frequency explained a greater percentage of variance of marathon finishing time (R2 = 20.20%) compared to the rest of the variables (R2 = 2.06% to 8.23%).

Multiple linear regressions models are shown in Table 11. In model 7, all the variables that remained as predictors of the marathon finishing time were included (exercise time, energy and sodium intake were excluded). Multicollinearity was not observed in this model (VIF = 1.22). All variables remained as predictors of the marathon finishing time when adjusting the model by age of participants (model 8). Nevertheless, when adjusting the model by sex of participants (model 9), CHO intake during competition ceased to be a predictor of marathon finishing time. So, in the final model (model 10) it is evident that marathon finishing time was higher in older, heavier subjects, with lower training frequency, higher fluid intake during competition, and among female participants. Multicollinearity was not observed in this model (VIF = 1.12).

**Table 11:** Multiple linear regression models in which marathon finishing time is considered the dependent variable, while sociodemographic and training characteristics and intra-competition intake are considered independent variables

	Model 1	Model 2	Model 3	Model 4	Model 5	Model 6	Model 7	Model 8	Model 9	Model 10
Intercept (α)	177.62^*c*^	176.52^*c*^	172.40^*c*^	178.47^*c*^	177.52^*c*^	177.92^*c*^	175.56^*c*^	130.53^*c*^	171.88^*c*^	128.56^*c*^
*Regression coefficient (β)*										
BMI (kg/m^2^)	3.33^*b*^	3.38^*b*^	1.16^*a*^	3.05^*b*^	3.09^*b*^	3.08^*b*^	3.13^*b*^	2.93^*a*^	4.43^*c*^	4.24^*c*^
Exercise frequency (s/w)	−9.03^*c*^	−8.50^*c*^	−8.58^*c*^	−8.00^*c*^	−7.96^*c*^	−7.95^*c*^	−7.73^*c*^	−7.15^*c*^	−7.73^*c*^	−7.00^*c*^
Exercise time (h/w)		−0.38								
Fluids (mL/kg)			0.43^*a*^	0.56^*b*^	0.55^*b*^	0.56^*b*^	0.46^*a*^	0.65^*b*^	0.57^*b*^	0.65^*c*^
Energy (kcal/h)				−0.08^*a*^	0.04					
Carbohydrates (g/h)					−0.50	−0.35^*a*^	−0.41^*b*^	−0.34^*a*^	−0.21	−0.21
Sodium (mg/kg)							0.49			
Age (yrs)								1.06^*b*^		0.97^*b*^
Sex									−35.37^*c*^	−34.18^*c*^
R^2^ (%)	24.35	24.46	27.12	29.61	29.89	29.86	30.64	34.41	40.36	44.18
SEE (min)	29.66	29.73	29.48	29.17	29.30	29.21	29.15	28.34	27.03	26.24
AIC	1541.74	1543.51	1483.19	1471.27	1464.07	1462.13	1462.43	1453.93	1439.50	1431.42
VIF	1.04	1.37	1.04	1.09	8.98	1.09	1.22	1.09	1.12	1.12

α, intercept; β, regression coefficient; R2, determination coefficient as percentage; *SEE* standard errors of estimate; *VIF* variance inflation factor; *AIC* Akaike’s information criterion. Coding for sex: 0 = female, 1 = male.

^a^*p* < 0.050, ^b^*p* < 0.010, ^c^*p* < 0.001. Models 8 to 10 were adjusted by age and sex

## Discussion

The main objective of this study was to describe the nutritional intake pre-during-post marathon race and its relation to the finishing time. The importance of fluids, CHO, and sodium intake, as well as the consumption of SS such as caffeine and their influence on athletic performance, is noteworthy. To our knowledge, this is the first study to examine pre-during-post race intake of several nutrients and their relationship with finishing time.

When analysing the intake of each nutrient across the different stages of the race, it was observed that pre-marathon fluid intake in our study group (7 ± 4 mL/kg) was close to the recommendations established by sports nutrition guidelines (5–7 mL/kg of body weight at least 4 h before the race) [25]. However, it was lower than the intake reported by trail runners (13 ± 20 mL/kg) and middle-distance triathletes (10 ± 7.5 mL/kg) in similar studies [17]. Since pre-race fluid intake may affect hydration status during the event, athletes should begin the race well-hydrated and consider specific race conditions [34]. It is recommended to carefully monitor hydration status before the race and individualize fluid intake based on personal and environmental factors. During the competition, participants in our study reported an average water consumption of 466 ± 279 mL/h, which aligns with ACSM recommendations [25]. This finding is consistent with previously published data in trail runners (447 ± 232 mL/h) (425 ± 250 mL/h) and middle-distance triathletes (422 ± 176 mL/h) [17], but higher than that reported for other marathon runners (354 ± 187 mL/h) [19]. In relation to post-competition sodium and fluid intake, which play a pivotal role in the rehydration process following exercise [35], there is significant variability in fluid consumption reported across different studies [17, 35]. Our findings showed higher post-race fluid intake (15 ± 8 mL/kg) compared to triathlon and trail running studies (11 ± 6 mL/kg and 10 ± 7 mL/kg, respectively) [17].

Regarding CHO consumption, current recommendations emphasize the importance of CHO intake for endurance sports [36]. CHO is one of the primary energy sources during prolonged exercise and can be consumed in various forms, including sports drinks, energy gels, sports bars, chews, and regular foods. However, most participants in the present study reported consuming CHO in liquid and semi-solid forms, such as energy gels [19, 37], rather than solid foods. This preference may be partially attributed to the ease of intake and better gastrointestinal tolerability during exercise [2]. A recent study showed that sports drinks were the most commonly consumed source of CHO among endurance athletes during training, indicating a preference for liquid forms [20]. Similar trends were observed in triathlon, where most competitors preferred liquid sources over solid foods [38]. In terms of the most consumed CHO source in the present study, the findings align with the literature, which consistently shows that energy gels are significantly more consumed than other forms [39, 40]. This may be explained by the practicality, easy digestibility, and portability that energy gels offer compared to other options.

In pre-competition, CHO loading is a strategy often used by marathon athletes before competition to maintain race speed for a longer time and delay the onset of fatigue. Moreover, the intake of 1–4 g/kg body mass of CHO 3–4 h before the race is recommended to top-off muscle glycogen content after the overnight fast given that most marathon races are early in the morning [2]. However, our study shows that marathon runners failed to meet CHO intake pre-competition recommendations (1 g ± 0.5 g/kg). A similar nutrition pattern has been reported in trail running (1 ± 1 g/kg) and middle-distance triathlon (1 ± 1.5 g/kg). Furthermore, it is well established that ingesting CHO during endurance competition can improve performance because it helps to maintain stable blood glucose levels and provides additional fuel for working muscles [4]. For long lasting events, carbohydrate intake during exercise is highly recommended, ranging between 60 and 90 g/h including multiple-transportable CHO sources depending on the exercise scenario (e.g., duration and ambient conditions) and individual tolerance [2, 41]. The current evidence suggests a wide variability of CHO intake during running events [16, 37, 42]. In trail races, previous research reported CHO consumptions of 15 g/h [16] and 23 g/h [37]. Pfeiffer et al. found an intake of 32 ± 15 g/h for other trail runners [18] and 35 ± 26 g/h among marathon runners [19], which is in concordance with our results (35 ± 17 g/h). On the other hand, when comparing with triathletes, their CHO intake was higher than in marathon athletes, as reported by Jiménez-Alfageme et al. (47 ± 19 g/h) [17] and Pfeiffer et al. (65 ± 25 g/h for middle-distance and 62 ± 26 g/h y 71 ± 25 g/h for long-distance triathlon) [19]. In post competition, guidelines suggest a CHO intake of 0.8-1 g/kg. Other authors [17] indicated that trail runners (1 ± 0.5 g/kg) and middle-distance triathletes (1 ± 0.8 g/kg) had a close CHO intake to those found in our work (1 ± 0.5 g/kg). Guidelines recommend an intake of 0.2–0.4 g/kg of protein to enhance the rate of muscle glycogen resynthesis [2, 43]. However, this value was not met in the analyzed sample, where the average protein intake was 0.1 ± 0.2 g/kg. The observed protein consumption was closer to that reported in middle-distance triathletes (0.2 ± 0.3 g/kg) but showed a more pronounced difference when compared to trail runners (0.4 ± 0.4 g/kg) [17]. Athletes should prioritize the post-competition period, which presents a strategic window for nutrient intake that can significantly influence subsequent endurance performance [9, 35, 43].

Moreover, to ameliorate physical performance decrements, sodium intake is recommended when exercise duration exceeds 2 h [24]. The American College of Sports Medicine (ACSM) suggests that runners consume between 300 and 600 mg of sodium per hour [25]. The purpose of sodium intake during prolonged exercise is to replace electrolytes and fluids lost through thermoregulatory sweating, maintain muscle contractility, enhance thirst stimulation, and reduce urine production [44]. The average sodium intake during competition was 192 ± 150 mg/h, which is below the recommended levels. In contrast, sodium ingestion was higher than that reported for other marathon runners (118 ± 87 mg/h) but consistent with findings suggesting similar sodium intakes among cyclists (208 ± 183 mg/h) [19]. Previous research has demonstrated significant variability in sodium intakes across different endurance sports, with trail runners exhibiting a range of 140–270 mg/h and middle-distance triathletes averaging 270 mg/h [16–18]. Collectively, these findings indicate that, despite the importance of sodium in electrolyte replacement, a substantial number of endurance athletes do not achieve the minimum recommended sodium intake.

Caffeine is one of the ergogenic aids most used by endurance athletes and doses of 3–6 mg/kg body mass before exercise have been shown to improve performance by 2–4% [14]. In our sample, the average pre-competition caffeine intake was 15 ± 1 mg/kg, which is significantly lower than the recommended dose. Despite this, the findings are not entirely consistent with previous research, which has identified caffeine as one of the most frequently consumed supplements in resistance sports [39, 40, 45]. However, the intake observed in our study was notably higher compared to middle-distance triathletes (0.3 ± 0.7 mg/kg) and trail runners (0.3 ± 0.4 mg/kg) [17]. During the competition, the average caffeine consumption in the present study was 57 ± 49 mg/h, which aligns with the recommendations suggesting that 50 mg/h of caffeine during exercise is sufficient to provide benefits for most athletes. It is important to note that the effectiveness of caffeine as an ergogenic aid may depend on individual biological responses, the timing of initial ingestion, and the duration of exercise [46, 47]. Interestingly, the caffeine intake observed in this study was higher than that reported for other marathon runners (23 ± 32 mg/h) [19], middle-distance triathletes (16 ± 26 mg/h), and trail runners (18 ± 27 mg/h) [17]. However, although caffeine at a dose of approximately 3 mg/kg during the recovery period has been shown to increase muscle glucose absorption by up to 66% [48], the caffeine intake in our study (0.5 ± 0.2 mg/kg) was well below the recommended levels. Nevertheless, our findings are consistent with other studies that reported even lower caffeine consumption among athletes [17].

The present study reveals that athletes who consumed less body-weight-relative fluids and more CHO per hour during the marathon were more likely to finish the competition in under 180 min. This finding suggests that higher CHO intake rates are associated with faster finishing times, overhydration may negatively affect performance. Although there is limited scientific evidence on the factors influencing competition finishing time, many studies have found no significant correlations between race time and factors such as age, years of training, or skeletal muscle mass (*P* > 0.05). However, fat mass and BMI have shown significant correlations with race time (*P* < 0.05) [49]. Otherwise, other authors concluded that no anthropometric variable was associated with total race time [49]. These results highlight the importance of educating athletes to meet sport nutrition guidelines during the competition and training the gut to improve fluid and CHO tolerance, which subsequently may lead to less GI discomfort and enhanced performance [46].

Regarding tolerance issues, a low prevalence of GI discomfort (13%) was reported during the marathon. This finding is consistent with previously published data (9%) by Martínez-Sanz et al. [50]. However, the literature reports higher prevalences among endurance athletes [17, 27, 51–53], demonstrating that these symptoms are a common issue faced by athletes. The low prevalence of GI complains in this study may be explained by the fact that runners consumed the lower limit of the suggested CHO intake range, and that GI problems tend to be more frequent in longer-duration races. Moreover, most of the runners included in our study reported having prepared in advance by testing the foods and/or supplements they would consume during the race, thereby minimizing gastrointestinal issues.Despite the fact that nutrition counselling is an important factor to support athletes’ performance, results showed that only 19% received specific guidance by a sport dietitian higher percentage (27%) was reported by trail runners in a previous study [16]. The primary source of nutrition counselling were the trainers, [45]. This suggests that athletes may perceive their coaches as having a substantial nutrition knowledge. Additionally, a significant proportion of runners (46%) did not receive any nutrition advice. Nevertheless, runners who followed a specific nutrition plan were more likely to finish the race in under 180 min, underscoring the importance of nutritional guidance in improving race performance.

This research had some limitations that should be discussed to improve its applicability to marathon runners. The first limitation is the relatively small number of participants, resulting in a low response rate relative to the total number of competing runners, as well as the sex-based heterogeneity. Nevertheless, the sample size was deemed statistically significant according to the principles applied. Additionally, the data collected in this study was self-reported and retrospective, which assumes the accuracy of participant’ responses and could potentially introduce inaccuracies, particularly regarding nutrient and supplement intake or GI issues. To mitigate this, dietary intake was assessed using a validated questionnaire, which enhanced the quality and precision of the data collected on nutritional intake, supplements, and GI complaints. Although the information relies on participant’ memory and may introduce some inaccuracies, athletes typically pay closer attention to their dietary intake compared to the general population, making them more likely to accurately recall their competition-related intake. Therefore, the findings of the present study may not be generalizable to other countries, cultures, or all endurance athletes.

## Conclusions

This is one of the first studies to comprehensively investigate the nutrient intakes pre, during and post-race among marathon runners. The intake of CHO, sodium and caffeine fell below current recommendations during the competition, while fluid intake was at the lower limit of the guidelines. Likewise, CHO intake after the competition met the recommendations whereas the recommended protein intake was not achieved. It is well established that CHO intake pre, during and post competition can influence exercise performance. The main source of CHO during the race was semi-liquid options, while liquid options were favoured post-competition and solid options were consumed in the hour leading up to the race.

Interestingly, the prevalence of GI discomfort was relatively low, indicating a high level of GI tolerance despite the small number of participants receiving specific nutritional recommendations from sport dietitians. Future research is needed to assess the relationship between dietary intake, GI issues and performance in larger sample sizes among marathon runners.

## Data Availability

All data supporting the findings of this study are available upon a reasonable request to corresponding authors.

## Abbreviations

IAAF: International Association of the Athletics Federations
MR: Marathon runners
CHO: Carbohydrates
ATP: Adenosine triphosphate
GI: Gastrointestinal
ACSM: American College of Sports Medicine
SD: Standard deviation
SS: Sport supplements
BMI: Body mass index
